# Synthesis and biological evaluation of novel 2-morpholino-4-anilinoquinoline derivatives as antitumor agents against HepG2 cell line†

**DOI:** 10.1039/d3ra07495a

**Published:** 2024-01-19

**Authors:** Ahmed Al-Sheikh, Malak A. Jaber, Hana'a Khalaf, Nour AlKhawaja, Duaa Abuarqoub

**Affiliations:** a Department of Pharmaceutical Medicinal Chemistry and Pharmacognosy, Faculty of Pharmacy and Medical Sciences, University of Petra Amman 11196 Jordan; b Department of Clinical Nutrition and Diets, Faculty of Pharmacy and Medical Sciences, University of Petra Amman 11196 Jordan; c Pharmaceutical Studies Center, Faculty of Pharmacy and Medical Sciences, University of Petra Amman 11196 Jordan; d Department of Pharmacology and Biomedical Sciences, Faculty of Pharmacy and Medical Sciences, University of Petra Amman 11196 Jordan duaa.abuarqoub@uop.edu.jo; e Cell Therapy Center, University of Jordan Amman 11942 Jordan

## Abstract

Cancer is a life-threatening illness all over the world, and developing anticancer treatments with high efficacy and low side effects remains a challenge. The quinoline ring structure has long been recognized as a flexible nucleus in the design and synthesis of physiologically active chemicals. In this study, five new 2-morpholino-4-anilinoquinoline compounds were synthesized and their biological anticancer potential against the HepG2 cell line was assessed. The compounds produced demonstrated varying responses against HepG2 cells, with compounds 3c, 3d, and 3e exhibiting the highest activity, with IC_50_ values of 11.42, 8.50, and 12.76 μM, respectively. It is a critical requirement that anticancer medications are able to selectively decrease cancer growth while not causing damage to normal cells. Compound 3e exhibited increased activity while maintaining adequate selectivity. It was also the most effective chemical against cell migration and adhesion, which could play an important role in drug resistance and cell metastasis. In total, the findings revealed good possibilities for anticancer therapy, suggesting a target for future development of anticancer medication.

## Abstract 2

Cancer is a life-threatening illness all over the world, and developing anticancer treatments with high efficacy and low side effects remains a challenge.

## Graphical abstract

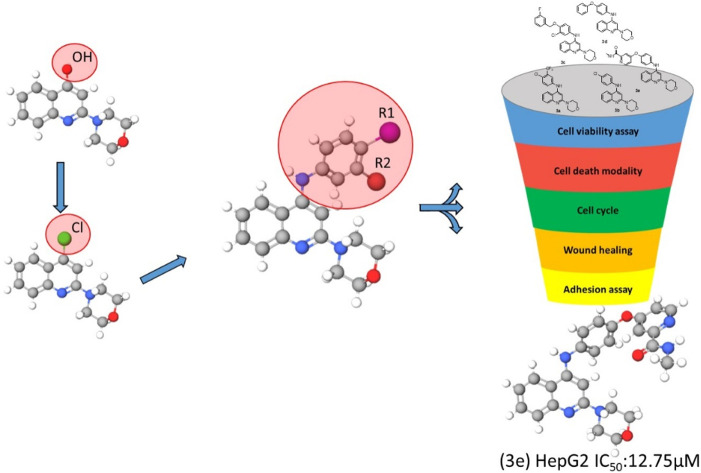

## Introduction

Cancer is characterized by abnormal cells that grow rapidly beyond their boundaries and that can spread to other organs, in a process called metastasis. Metastasis is the primary cause of death from cancer, making cancer the leading cause of death worldwide.^1^ For a long time, cytotoxic therapy has been the gold standard in cancer treatment.^2^ However, inconsistency in the therapeutic response and the low safety profile have led to the development of novel cancer-targeting techniques, which has increased the weaponry against numerous cancers.^2,3^

Quinoline refers to a group of chemical compounds from the aromatic heterocyclic family that are characterized by a double-ring structure composed of a benzene ring and a pyridine ring fused to form a double-ring structure.^4^ A quinoline moiety is present in numerous natural compounds and has a wide range of biological activity.^5^ In addition to having anticonvulsant, cardiotonic, anti-inflammatory, and analgesic properties, quinoline has been shown to be active against bacteria, fungi, parasites, worms, and other organisms.^5,6^ Quinoline derivatives possess high potential anticancer activity through a variety of mechanisms.^5,7–9^

**Fig. 1:**
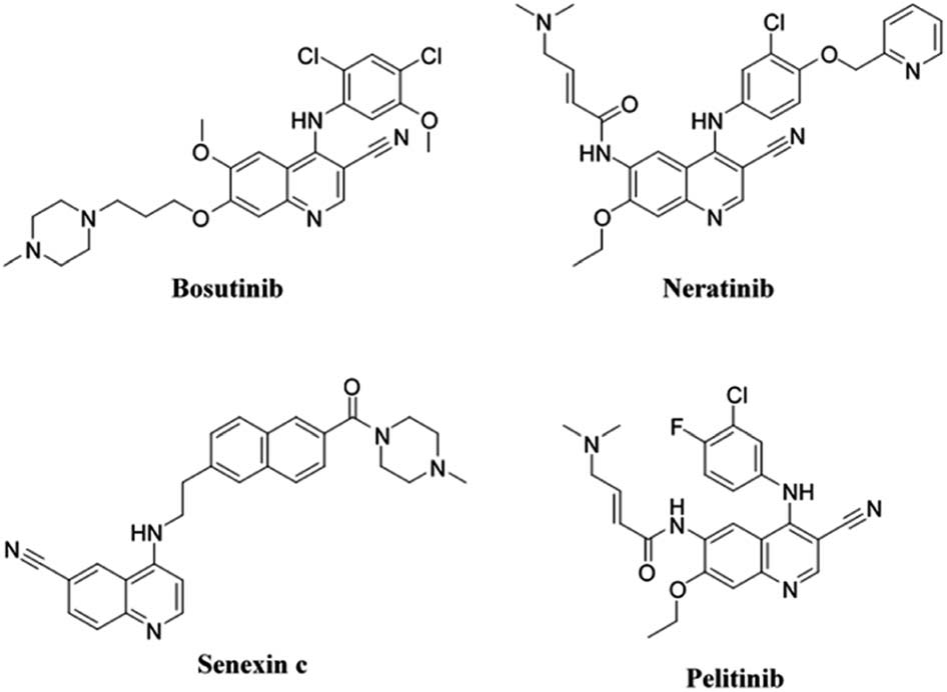
Some of the quinoline-based derivatives approved for therapy or under clinical trial for treatment of cancer: bosutinib, neratinib, senexin c, and pelitinib.

Several derivatives of 4-aminoquinoline have been approved in therapy or are undergoing clinical trial for the treatment of cancer (Fig. 1). The anticancer medications bosutinib and neratinib, both of which have been approved for medical use, are notable examples of this class of compounds. Bosutinib is one of five tyrosine kinase inhibitors^10^ indicated for first-line treatment of chronic myelogenous leukemia, due to its ability to inhibit Bcr-Abl and Src kinases.^11^ Neratinib, on the other hand, has been licensed for the treatment of breast cancer.^12^ Neratinib binds to epidermal growth factor receptor (EGFR) and human epidermal growth factor receptor 2 and 4 (HER2 and HER4, respectively) and suppresses them permanently,^13^ by preventing the tyrosine residues on the receptor from becoming autophosphorylated, and this lowers oncogenic signaling *via* the protein kinase B and mitogen-activated protein kinase pathways.^14^ Furthermore, senexins are powerful and selective quinazoline inhibitors of CDK8/19 mediator kinases, which function as novel resistance-preventing drugs for the treatment of cancer.^15,16^ Additionally, clinical trials are currently being conducted on the quinoline derivative pelitinib (EKB-569) for the treatment of colorectal and lung cancer. It has been described as a potent selective, and permanent EGFR inhibitor.^17,18^Fig. 1 provides a more detailed look at the chemical structures of these described drugs.

Many researchers have investigated various quinoline derivatives as potential anticancer drugs. According to a study by Iqbal *et al.*, various new compounds comprising isoquinoline derivatives, quinoline-4-carboxylic acid derivatives, and 4-quinolone derivatives showed specific cytotoxicity against malignant cells.^7^ Using a xenografted model on nude mice, Zhou *et al.* showed that novel quinoline derivatives possess anticancer activity by modulating the extracellular matrix of cancer cells. This was achieved by downregulating lumican, a small leucine-rich proteoglycan linked to several cancer types, which is linked to pro-tumorigenic or antitumorigenic activity.^19^ Another group concluded that IND-2, a quinoline derivative, can decrease the proliferation of prostate cancer cells, and so could be utilized to control their growth, proliferation, and metastasis.^20^

**Fig. 2:**
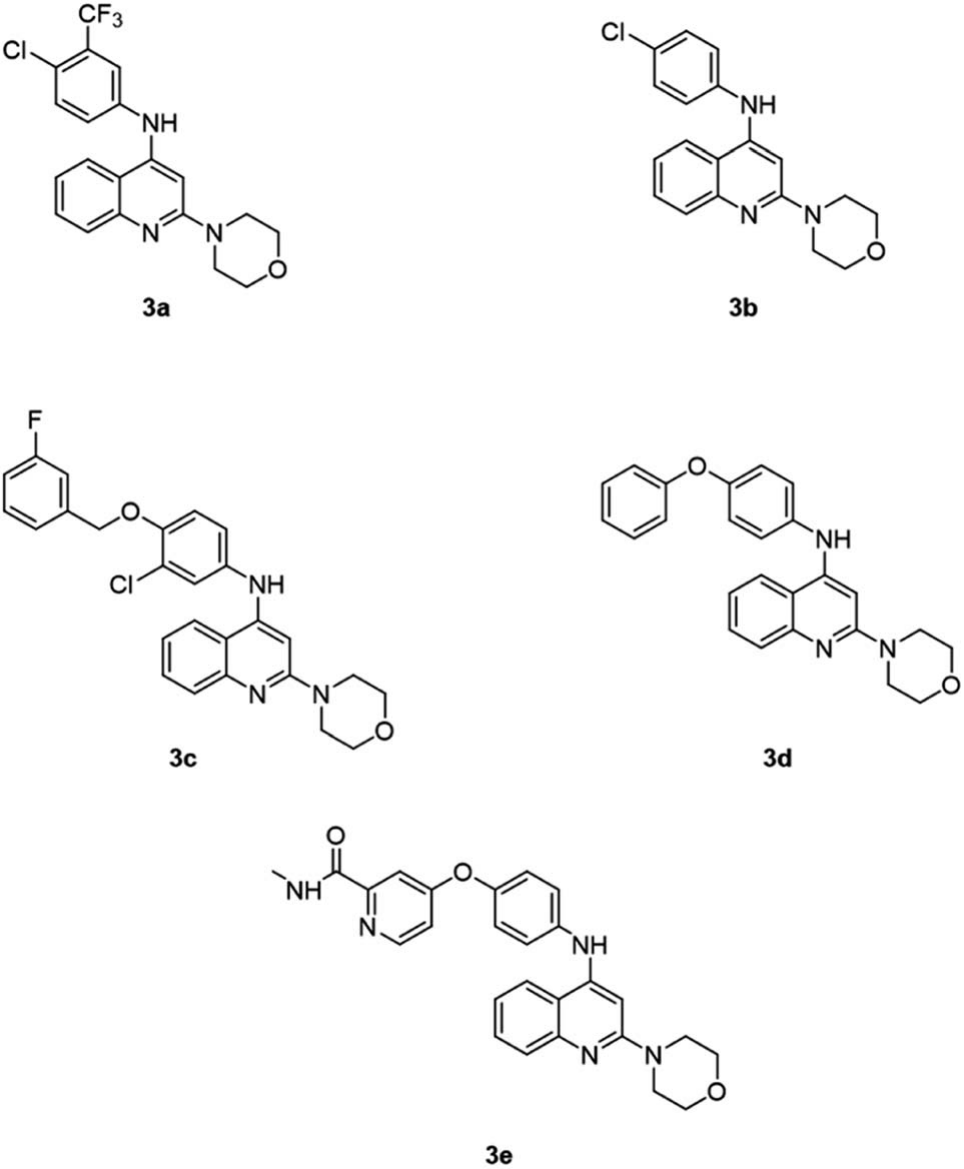
Chemical structures of quinoline-based derivatives studied in this work.

Due to the broad range of biological and pharmacological properties of quinoline and its derivatives, they are considered an important class of compounds for new drug development. As a result, many new therapeutic agents have been developed by using quinoline nuclei, through numerous synthetic routes.^21^ This article covers the synthesis, as well as the biological activity against the HepG2 cancer cell line, of the new quinoline derivatives 3a–3e (Fig. 2).

## Methodology

### Synthesis of quinoline derivatives

**Scheme 1:**
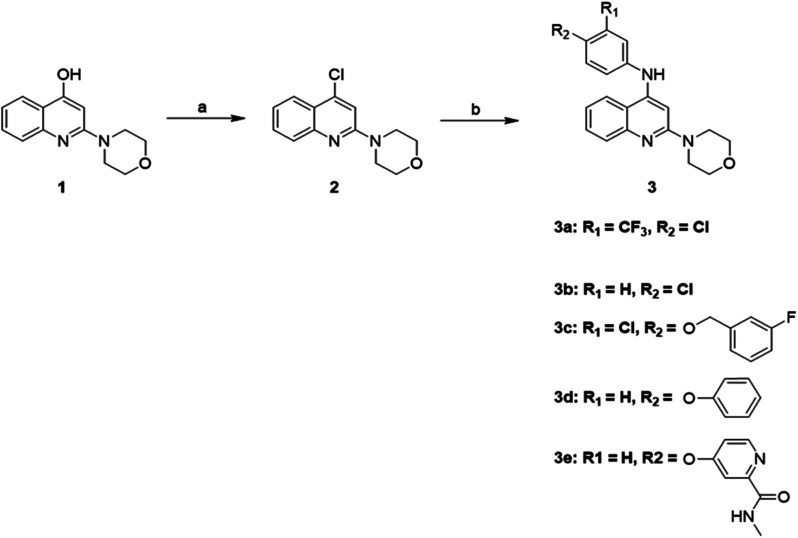
Synthesis of 2-morpholino-4-anilinoquinoline derivatives using 2-morpholinoquinolin-4-ol, 1, through the formation of 4-chloro-2-morpholinoquinoline, 2. Reagents and conditions: (a) POCl_3_, 90 °C; (b) corresponding aniline, ethanol, reflux.

As illustrated in Scheme 1, a variety of 2-morpholino-4-anilinoquinoline derivatives were synthesized starting from 2-morpholinoquinolin-4-ol, 1. Chlorination of 1 with phosphorus oxychloride resulted in 4-chloro-2-morpholinoquinoline, 2. Transformation of 2 to 2-morpholino-4-anilinoquinoline derivatives 3a–3e was carried out by substituting the chlorine atom with the corresponding aniline.

### Cell viability assay

A viability screening test was performed to evaluate whether the synthesized 2-morpholino-4-anilinoquinoline derivatives have effects on cell proliferation or have direct cytotoxic effects on the HepG2 cell line. The cell viability test was carried out using the MTT reagent (3-(4,5-dimethyl thiazol-2yl)-2,5-diphenyltetrazolium bromide) (Promega, Madison, WI, USA), as described below. A total of 5 × 10^3^ HepG2 cells (ATCC, USA) were seeded into a 96-well plate (SPL Life Sciences Co., Ltd, Gyeonggi-do, Korea) in triplicate and allowed to attach for 24 hours, before being treated with one of the 2-morpholino-4-anilinoquinoline derivatives at two concentrations, of 3 and 30 μM, for 72 hours at 37 °C in a 5% CO_2_ incubator. DMEM-HG supplemented with 10% fetal bovine serum (FBS), 1% l-glutamine, and 1% penicillin/streptomycin only (Gibco, Carlsbad, CA, USA) was used as the blank control. Following incubation, the old medium was replaced with 100 μL of fresh medium, 15 μL of MTT (5 mg mL^−1^) was added to each well, and the plates were incubated at 37 °C for 3 hours. After that, the formazan crystals formed were dissolved by the addition of 50 μL per well dimethyl sulfoxide (DMSO), and the optical density (OD) was measured at 570 nm using a Glomax plate reader (Promega, Madison, WI, USA). The percentage cell viability was calculated using the following equation.^22^Cell viability (%) = sample optical density/control optical density × 100

The percentage cell survival of the top three most potent compounds was then studied at various concentrations, ranging from 50 to 1.56 μM in a two-fold serial dilution under the same conditions as described above. Then, the half maximal inhibitory concentration (IC_50_) value of each compound was calculated using nonlinear regression (variable slope, four parameters) of the log concentration as inhibition percentage values, using GraphPad Prism 9.0.

### Apoptosis/necrosis cell death modality

The mode of cell death was assessed by flow cytometry *via* the annexin V/propidium iodide (PI) apoptosis kit (Abcam, UK) on two different cell lines: cancer cells (HepG2) and normal mouse cells (fibroblast, NIH3T3). Briefly, cells from the selected cell lines were seeded into a six-well tissue culture plate (SPL, Gyeonggi-do, Korea) at a density of 2 × 10^5^ cells per well and treated for 72 hours with one of the 2-morpholino-4-anilinoquinoline derivative at two concentrations (IC_50_ and 20 μM). Untreated wells served as a control. Then, the treated cells were harvested and digested with trypsin-EDTA (Biowest, France), washed with phosphate-buffered saline (PBS) (Gibco, Carlsbad, CA, USA), and centrifuged at 300×*g* for 5 min. The cell pellets were then stained with annexin V/PI according to the manufacturer's instructions (Invitrogen, Waltham, MA, USA). The samples were analyzed immediately with a flow cytometer using FACS DIVA v8 software and the FACSCanto™ II system (BD Biosciences, Franklin Lakes, NJ, USA).

### Cell cycle

PI staining was used in flow cytometric analysis to evaluate the impact of selected compounds on the cell cycle analysis of HepG2 cells. First, HepG2 cells were seeded into a six-well tissue culture plate (SPL, Gyeonggi-do, Korea) at a density of 2 × 10^5^ cells per well. Cells were treated for 72 hours with one of the 2-morpholino-4-anilinoquinoline derivatives at two concentrations (IC_50_ and 20 μM) after they had reached confluence, or they were left untreated to act as a control. Then, the treated cells were washed twice with 100 μL of ice-cold PBS (Gibco, Carlsbad, CA, USA) and centrifuged at 300×*g* for 5 min after each wash cycle. Cells were resuspended by adding 100 μL of ice-cold PBS and 300 μL of PI (R&D systems kit, USA) per tube. After that, samples were kept in the dark at room temperature for 30 min. Cells were further diluted by the addition of 200 μL of ice-cold PBS/flow tube and samples were examined by flow cytometry using the FACSCanto™ II system. Data acquisition and analysis were performed using BD FACSDiva™ v8 (BD Biosciences, USA) and Flowlogic™ 7.3 (Melbourne, Australia).

### Wound healing (scratch assay)

HepG2 cells were planted at a density of 2 × 10^5^ cells per well in six-well plates with culture media and allowed to fully confluence as a monolayer. HepG2 cells were treated with serum-free starvation medium for 24 hours before application of a wound. A wound (scratch) was inflicted through 100% confluent cells using a 200 μL pipette tip. Cell debris was then removed by rinsing the area with PBS. Injured cells were treated with one of the 2-morpholino-4-anilinoquinoline derivatives at two concentrations (IC_50_ and 20 μM) along with a blank and positive control for 48 hours. Using a phase-contrast microscope (Zeiss, Oberkochen, Germany), progression of the wound closure was monitored, and images of the scratch were acquired at two separate time points: before scratching at 0 hours and 48 hours after wound infliction. For calculation of wound closure (%), measurements were taken at 0 hours and 48 hours, and the calculations were as follows:Wound closure (%) = 100 − (distance at 48 hours − distance at 0 hours)/(distance at 0 hours) × 100%

### Adhesion assay

The adhesion assay was performed on HepG2 cells cultured in DMEM-HG containing 10% FBS at 80% confluence in a 96-well plate coated with fibronectin (Merck, Germany). Cells were initially washed with PBS and resuspended at a density of around 1 × 10^5^ cells per mL in serum-free medium. Subsequently, 100 μL of the cell suspension was added to each pre-coated well, with the addition of appropriate quantities of test compounds at IC_50_ and 20 μM, and incubated for 24 hours at 37 °C in 5% CO_2_. After treatment, the coated plate was blocked with 100 μL of 0.2% bovine serum albumin (Sigma, USA) for 24 hours at room temperature. Then, non-adherent cells were aspirated along with the medium, and adherent cells were washed using 100 μL Dulbecco's PBS per well, three times. The adhering cells were stained with 0.1% crystal violet for 30 min, which binds to the DNA and protein of the cell. Then, 10% acetic acid was added to solubilize the dye, and the OD was measured at 595 nm on a multiplate reader (Glomax, Promega, Madison, WI, USA).

## Experimental section

### Synthetic procedures

All chemical reagents and solvents were obtained commercially and were utilized without further purification. All NMR spectra were acquired in deuterated DMSO using a Bruker spectrometer at 500 MHz. High-resolution mass spectra (HRMS) were obtained using an electrospray ionization technique on a Bruker Impact II mass spectrometer in positive-ion mode at 2500 V.

The compound 2-morpholinoquinoline, 1, was synthesized according to a previously published procedure.^23^

#### 2-Morpholino-4-chloroquinoline (2)

A solution of 2-morpholinoquinoline 1 (0.5 g, 2.17 mmol) in phosphorus oxychloride (1.5 mL) was heated to 95 °C for 3 h. After cooling to room temperature, it was poured onto crushed ice (40 mL). The solution was cooled to 0 °C and neutralized by dropwise addition of a saturated solution of NaHCO_3_. The mixture was extracted with dichloromethane (2 × 23 mL). The combined organic layers were dried over Na_2_SO_4_, filtered, and concentrated under vacuum to yield 2 as a light yellow solid in 67% yield. ^1^H NMR (500 MHz, CDCl_3_) *δ*: 8.02–7.33 (m, 4H), 7.07 (s, 1H), 3.85 (m, 4H), 3.70 (m, 4H). ^13^C NMR (126 MHz, CDCl_3_) *δ*: 157.03, 148.37, 143.55, 130.65, 127.00, 123.94, 123.40, 121.48, 109.25, 66.78, 45.53. HRMS ESI, ([M + H]^+^): calculated *m*/*z* 249.0789; found, 249.0804 Fig. S1.†

#### General procedure for the synthesis of 2-morpholino-4-anilinoquinoline (3a–3e)

To a solution of 2 (0.2 g, 0.87 mmol) in ethanol (10 mL) was added the appropriate aniline (1.7 mmol). The resulting mixture was refluxed overnight. The ethanol was evaporated under vacuum. The resulting residue was washed with acetone and filtered to yield (3a–3e).

#### *N*-(4-Chloro-3-(trifluoromethyl)phenyl)-2-morpholinoquinolin-4-amine (3a)

Yield: 42%. ^1^H NMR (500 MHz, DMSO-d_6_) *δ* 12.84 (s, 1H), 10.32 (s, 1H), 8.56–7.48 (m, 7H), 6.47 (s, 1H), 3.72 (m, 8H). ^13^C NMR (126 MHz, DMSO-d_6_) *δ*: 153.87, 151.83, 139.08, 133.26, 132.94, 128.66, 127.94, 126.40, 124.73, 124.17, 123.68, 123.18, 122.00, 115.19, 87.96, 66.00, 47.18. HRMS ESI, ([M + H]^+^): calculated *m*/*z* 408.1085; found, 408.1090.

#### *N*-(4-Chlorophenyl)-2-morpholinoquinolin-4-amine (3b)

Yield: 93%. ^1^H NMR (500 MHz, DMSO-d_6_) *δ*: 12.87 (s, 1H), 10.23 (s, 1H), 8.61–7.79 (m, 8H), 6.21 (s, 1H), 3.71 (br. s, 8H). ^13^C NMR (126 MHz, DMSO-d_6_) *δ*: 153.66, 152.81, 138.46, 137.70, 133.10, 130.37, 130.12, 126.70, 124.87, 123.61, 119.15, 114.84, 86.12, 65.94, 47.18. HRMS ESI, ([M + H]^+^): calculated *m*/*z* 340.1211; found, 340.1213.

#### *N*-(4-(3-Fluorobenzyloxy)-3-chlorophenyl)-2-morpholinoquinolin-4-amine (3c)

Yield: 76%. ^1^H NMR (500 MHz, DMSO-d_6_) *δ*: 8.65 (s, 1H), 8.11 (s, 1H), 7.52–7.18 (m, 10H), 6.38 (s, 1H), 5.23 (s, 2H), 3.66 (s, 4H), 3.44 (s, 4H). ^13^C NMR (126 MHz, DMSO-d_6_) *δ*: 163.65, 161.71, 158.55, 150.23, 149.75, 140.06, 135.28, 131.04, 130.11, 126.89, 125.16, 123.85, 123.12, 122.54, 122.17, 121.66, 116.54, 115.65, 115.09, 114.46, 88.39, 69.97, 66.51, 45.68. HRMS ESI, ([M + H]^+^): calculated *m*/*z* 464.1535; found, 464.1550.

#### 2-Morpholino-*N*-(4-phenoxyphenyl)quinolin-4-amine (3d)

Yield: 30%. ^1^H NMR (500 MHz, DMSO-d_6_) *δ* 12.77 (s, 1H), 10.11 (s, 1H), 8.59–7.09 (m, 13H), 6.12 (s, 1H), 3.72–3.38 (m, 8H). ^13^C NMR (126 MHz, DMSO-d_6_) *δ* 156.85, 155.20, 153.30, 138.48, 133.71, 133.01, 130.65, 127.13, 124.80, 124.26, 123.44, 119.95, 119.36, 114.70, 85.35, 65.94, 47.08. HRMS ESI, ([M + H]^+^): calculated *m*/*z* 398.1863; found, 398.1887.

#### 4-(4-(2-Morpholinoquinolin-4-ylamino)phenoxy)-*N*-methylpyridine-2-carboxamide (3e)

Yield: 24%. ^1^H NMR (500 MHz, DMSO-d_6_) *δ*: 12.87 (s, 1H), 10.18 (s, 1H), 8.79–7.24 (m, 11H), 6.18 (s, 1H), 3.73–3.69 (m, 8H), 2.79 (s, 3H). ^13^C NMR (126 MHz, DMSO-d_6_) *δ*: 165.99, 164.24, 153.94, 152.98, 151.44, 151.41, 151.02, 136.46, 132.87, 127.38, 124.70, 124.65, 123.47, 122.77, 114.92, 109.44, 86.00, 66.02, 47.07, 26.50. HRMS ESI, ([M + H]^+^): calculated *m*/*z* 456.2030; found, 456.2034.

## Results

### Cell viability

**Fig. 3:**
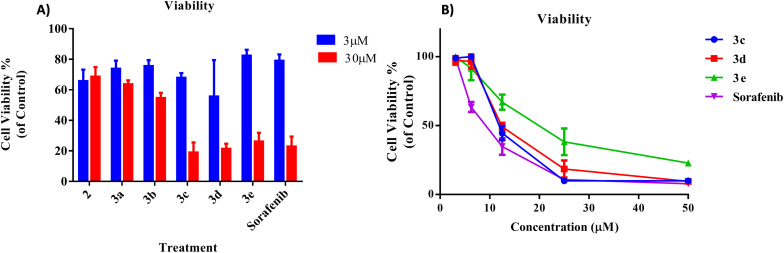
*In vitro* cell viability assay of (A) all synthesized 2-morpholino-4-anilinoquinoline derivatives at concentrations of 3 and 30 μM and (B) top three most potent compounds on human cancer cell line HepG2 using MTT reagent after 72 hours incubation at 37 °C in a 5% CO_2_ incubator. The cell viability was calculated based on the OD at 570 nm of treated samples in comparison to the control OD, which was considered as 100%.

The results for the inhibitory analysis of the 2-morpholino-4-anilinoquinoline derivatives are presented in Fig. 3. As illustrated in Fig. 3A, the cell viability of synthesized compounds to HepG2 cells was variable. At low concentrations (3 μM), most compounds, including sorafenib (positive control), exhibited minimal cytotoxicity, ranging from 40% for compound 3d to 20% for compound 3e. Compounds 3c, 3d, and 3e had the maximum cytotoxicity at higher concentrations (30 μM) compared to the other compounds.

As can be seen in Fig. 3B, the viability of later compounds decreases with increasing concentration. Sorafenib showed the highest potency in the cell viability testing, with an IC_50_ of 5.2 ± 0.07 μM, followed by 3d (8.5 ± 0.08), 3c (11.42 ± 0.01), and 3e (12.76 ± 0.07 μM).

### Apoptosis/necrosis cell death modality

To assess the death modality of selected compounds, an apoptosis/necrosis assay was performed after treating cells (cancer cells: HePG2 and normal cells: fibroblast NIH3T3) with one of the selected compounds for 72 hours.

**Fig. 4:**
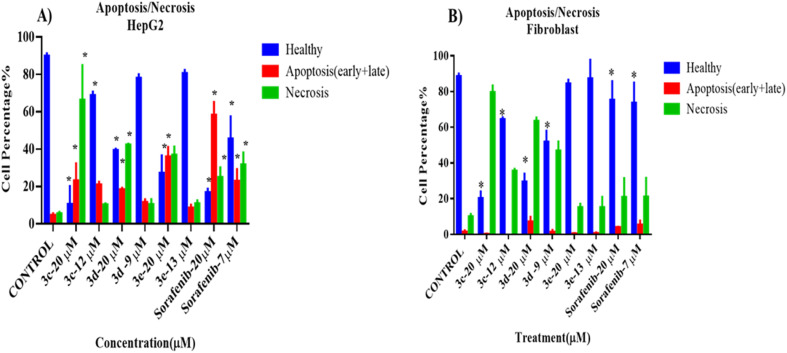
Cell death modality assay of 2-morpholino-4-anilinoquinoline derivatives on (A) HepG2 and (B) fibroblast (NIH3T3) cells, analyzed by flow cytometry after treatment with annexin/PI stain. Data in (A) and (B) are shown as mean ± SD of triplicates compared with the untreated control. One-way ANOVA analysis using Tukey's post *hoc* test was used to indicate significance. *p value ≤ 0.05, compared with control.

The results of the apoptosis/necrosis assay are shown in Fig. 4. All selected compounds at high concentration (20 μM, higher than IC_50_) were cytotoxic against HepG2cells, as the percentage of healthy cells decreased significantly compared to the control, untreated group (*p* < 0.05). In addition, necrosis was the major cellular death modality responsible for the cytotoxicity upon treatment with these compounds, rather than apoptosis, which was induced by the positive control. Also, only 3c and sorafenib (positive control) had cytotoxic effects against HepG2 at their IC_50_, as the proportion of healthy cells was dramatically reduced (*p* < 0.05). No difference was seen after cells were exposed to IC_50_ doses of 3d and 3e, respectively.

Interestingly, compound 3e did not show any cytotoxic effects on the treated fibroblasts at both high and low concentrations, while 3c, 3d, and sorafenib showed a significant decrease in the number of healthy cells and an increase in the number of necrotic cells when compared to the control, untreated cells. Representative data obtained from flow cytometric dot plot analysis of cell death modality (apoptosis/necrosis) are listed in Fig. S2.†

### Cell cycle

**Fig. 5:**
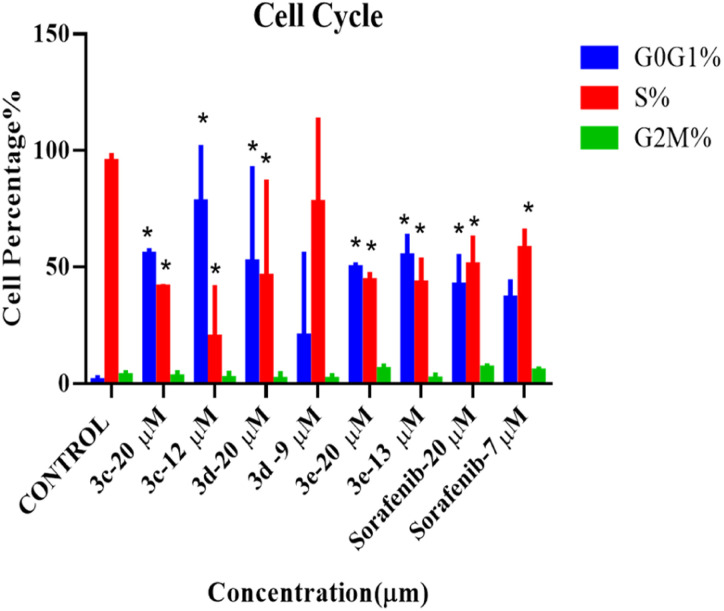
Cell cycle analysis of HepG2 cells treated with 2-morpholino-4-anilinoquinoline derivatives at two concentrations (IC_50_ and 20 μM) for 72 hours before harvest. Histogram plots displaying percentage of G0G1 (blue), S phase (red), and G2M (green) cell populations. Error bars represent the mean ± SEM of three biologically independent experiments. One-way ANOVA analysis using Tukey's post *hoc* test was used to indicate significance. *p value ≤ 0.05, compared with control.

According to the results from the cell cycle study shown in Fig. 5, the cell cycle phases of treated cells differ significantly from those of the control group. Under all treatment conditions, there was a noticeable increase in the number of cells in the G0G1 phase. Halting of the cell cycle in the G0G1 phase was accompanied by a drop in the percentage of cells in the S phase, indicating that these chemicals can induce cell cycle arrest in the G0G1 phase. In addition, no alterations were observed in the G2M phases. As a result, these compounds, except for compound 3d, at their IC_50_ can inhibit HepG2 cell proliferation during the G0/G1 phase. Histograms derived from flow cytometry analysis are listed in Fig. S3.†

### Wound healing (scratch assay)

**Fig. 6:**
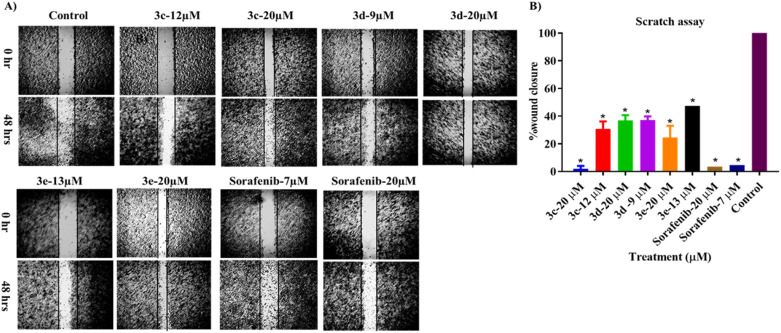
Effect of the tested compounds on the migration of HepG2 cancer cell line presented as (A) phase-contrast images acquired before scratching at 0 hours and 48 hours after wound infliction, and (B) percentage of wound closure after 48 hours of treatment. One-way ANOVA analysis using Tukey's post *hoc* test was used to indicate significance. *p value ≤ 0.05, compared with control.

A scratch experiment was conducted to assess the ability of the tested compounds to prevent cancer cells from migrating. According to the data in Fig. 6, after 48 hours of incubation with the test compounds, more cells migrated into the scratched area in the control group than in the treated cells. Nevertheless, all compounds significantly reduced the ability of the treated cells to migrate, in a dose-dependent manner. However, among the tested derivatives, compound 3c at 20 μm had the strongest potential to decrease or prevent HepG2 cell migration when compared with sorafenib.

### Adhesion assay

**Fig. 7:**
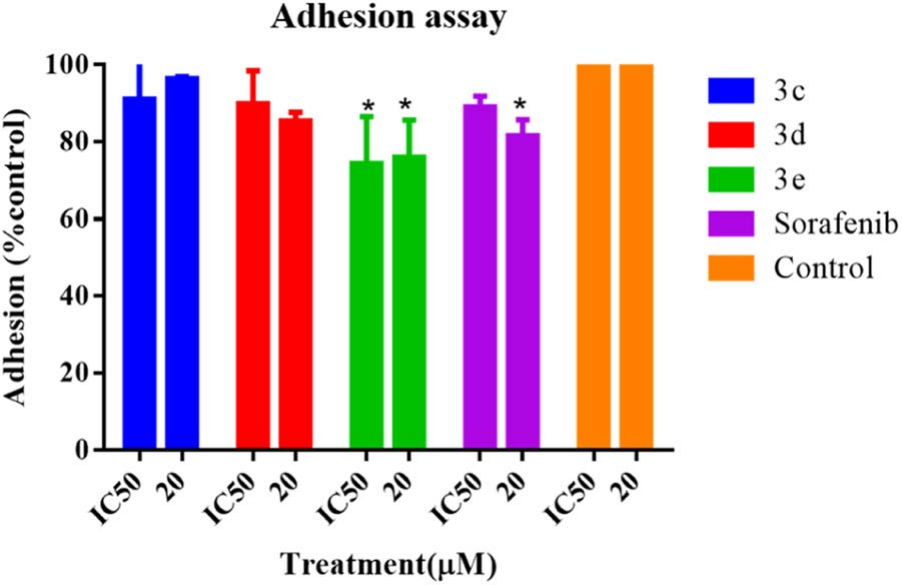
Adhesion assay of HepG2 cells after treatment with selected compounds at IC_50_ and 20 μM for 24 hours. Error bars represent the mean ± SEM of three biologically independent experiments. One-way ANOVA analysis using Tukey's post *hoc* test was used to indicate significance. *p value ≤ 0.05, compared with control.

An adhesion assay was carried out to examine the impact of the selected compounds on the adhesion capacity of the treated cells. When compared to the untreated control group, cells treated with 3e at both IC_50_ and 20 μM, as well as the positive control (sorafenib) at 20 μM, dramatically reduced the adhesion potential of HepG2 cells, as seen in Fig. 7, whereas HepG2 cell adhesion was not prevented or diminished by any component of 3c or 3d.

## Discussion

Cancer is a disease that is caused by anomalies in the cell cycle, which result in uncontrolled cell division of the altered cells. Cancerous diseases are, without doubt, one of the world's most serious health issues. Quinoline derivatives are one of the most active groups of nitrogen-containing heterocyclic aromatic chemicals. The activity of these compounds is mostly determined by the characteristics of the substituents, as well as by their presence and position on one of the cyclic molecules. Instead of focusing on DNA replication or cell division, these compounds are known to target cell signaling transduction systems and are considered to be a relatively new strategy in cancer therapy. Here, for the first time, the effects of five morpholino-4-anilinoquinoline derivatives on the HepG2 cell line were studied.

Several targeted alterations at the aniline *para* position, or both the *para* and *meta* positions, of the 2-morpholino-4-anilinoquinoline core were produced and tested for anticancer efficacy. The cytotoxicity of the utilized intermediate 2 was negligible at 30 μM and comparable to the cytotoxicity of compounds 3a and 3b. Except for compound 2, these compounds feature minor alterations at the *para* and/or *meta* positions of the aniline cycle. Compounds 3c, 3d, and 3e, on the other hand, exhibited numerous larger substitutions at the same location. As a result, increasing the variety of the substitution resulted in comparable cytotoxic activity to sorafenib, by occupying a distinct chemical space.

Cell death modality testing on HepG2 and fibroblast cell lines found that compounds 3c and 3d exhibited the highest cytotoxic activity against both cell lines, demonstrating non-selective action toward cancer cells. While compound 3e showed substantial activity against HepG2 cells at 20 μM (but not at its IC_50_), it showed insignificant cytotoxicity against fibroblasts, indicating its selectivity for cancer cells and safety in comparison to other compounds. In comparison to sorafenib, compound 3e exhibited weaker activity against HepG2 cells at 20 μM but higher selectivity.

**Fig. 8:**
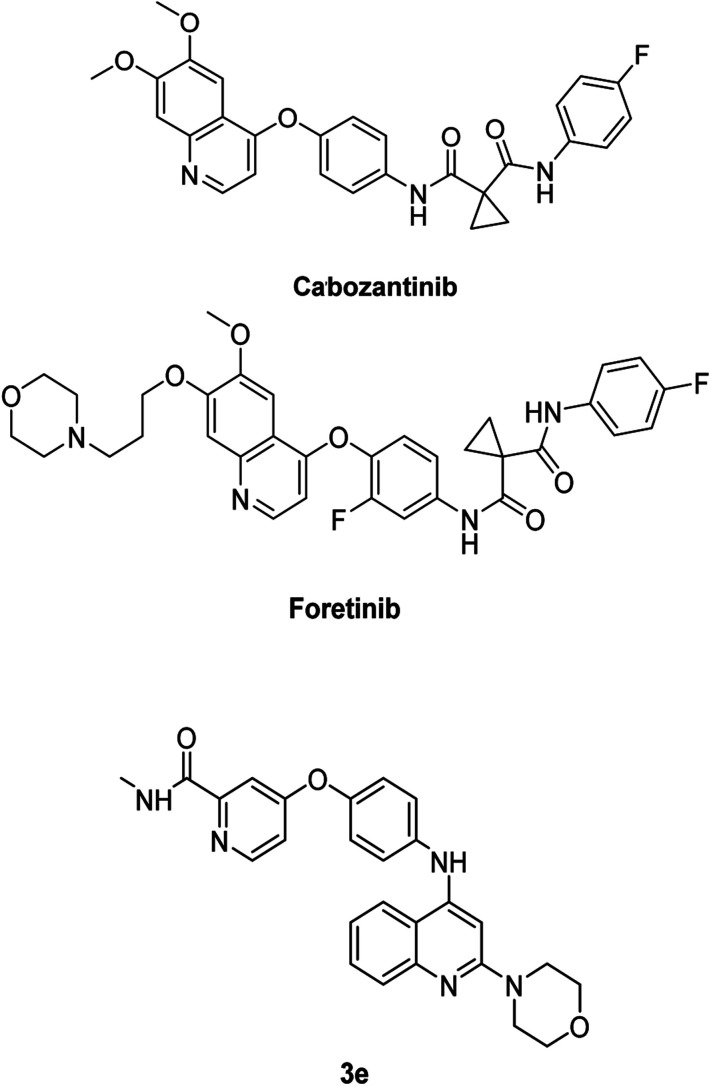
Structure of quinoline inhibitors: cabozantinib, foretinib, and 3e.

Epithelial–mesenchymal transition, or EMT, is the highly dynamic process involved in the conversion of epithelial cells into mesenchymal cells, multipotent stem cells stimulated by a network of signaling pathways, resulting in changes in cellular morphology, growth, and motility.^24–26^ MET protein is a receptor protein-tyrosine kinase for hepatocyte growth factor produced by stromal and mesenchymal cells that regulates cell growth, morphogenesis, and motility. MET overexpression has been recognized in several human malignancies, and numerous quinoline-based derivatives are thought to be MET inhibitors, as they interact with the kinase domain of the receptor in the cytosol.^27^ Previous structure–activity relationship analyses have shown that the anticancer activity of quinoline derivatives is linked to three crucial aspects when using cabozantinib and foretinib as lead drugs for the synthesis of novel quinoline derivatives preferentially acting on MET: the existence of donor and/or acceptor hydrogen bond groups, as well as at least one amide, and the presence of five atoms between the 4-phenoxyquinoline moiety and the aromatic one at C4.^28^ These broad characteristics can also be detected in the structure of cabozantinib, foretinib, and compound 3e, as shown in Fig. 8.

Furthermore, vascular and epidermal growth factor receptors (VGFR and EGFR, respectively) belong to a family of tyrosine kinase receptors associated with MET oncogenic pathways.^29^ GFR and/or VEGFR recruit rat sarcoma (RAS) proteins, which activate other proteins, which in turn activate genes essential in cell growth and survival. RAS signaling that is excessive can eventually lead to cancer. The 4-aniline quinolones and quinazoline scaffold have long been recognized as a classic receptor inhibitor.^29–32^

Additionally, as the cell population of HepG2 cells at the G0G1 phase increased, this suggests that the quinoline derivatives may encourage cancer cells to accumulate at the G0G1 phase and so they cannot complete the regular cell cycle as usual, resulting in cancer cell growth suppression. 8-(4-(Trifluoromethyl)benzyloxy)-1,2,3,4-tetrahydro-2-methylquinoline has been shown to dramatically decrease esophageal squamous cell cancer growth by downregulating COX-2 and PGE2 synthesis.^33^ Other quinoline compounds have been shown to disrupt genes involved in immune regulation and inflammation.^34^

Furthermore, the capacity of tested compounds to impact migratory cells could reveal the ability of these derivatives to antagonize the antitumorigenic actions of lumican. Lumican is a non-collagenous extracellular matrix protein that belongs to class II of the short leucine-rich proteoglycan family (SLRPs).^35,36^ Lumican is overexpressed in a variety of cancers, and it is thought to be responsible for promoting cancer cell migration, invasion, and proliferation, while its downregulation is important in reducing cancer cell migration and invasion.^37,38^ The novel quinoline derivative (compound 91b1) developed by Yuan Zhou demonstrated antitumor efficacy in nude mice *via* downregulation of lumican. In addition, Toshiyuki Ishiwata's group found that lumican plays a vital function in the prevention of human embryonic kidney 293 cell attachment,^39^ as well as resulting in morphological changes to cell shape and appearance, and hence altering cell adhesion ability.^40^ When compared to other compounds, compound 3e can considerably limit cell migration and decrease cell adhesion. Hence this compound provides a promising candidate for anticancer therapy.

## Conclusions

In conclusion, five 2-morpholino-4-anilinoquinoline compounds were effectively synthesized and biologically assessed. These compounds, with varied C4 aniline moieties, showed potent anticancer activity against HepG2 cancer cells. The chemicals were able to cause G0G1 cell cycle arrest and eventually limit HepG2 cell proliferation. Among these compounds, compounds 3c, 3d, and 3e displayed the highest cytotoxic activity against both cell lines; however, only compound 3e exhibited greater selectivity against cancer cells, demonstrating its safety in comparison to the other compounds. Furthermore, compound 3e can significantly inhibit cell migration and decrease cell adhesion. As a result, this chemical is a good option for anticancer therapy. Overall, our analysis identified several novel quinoline analogs that merit further investigation as possible anticancer drugs.

## Author contributions

A. A., M. J., and D. A. designed and planned the experiments. A. A. and N. A. worked out the chemistry part. D. A. and H. K. worked out the biological part. M. J. took the lead in writing the manuscript. All authors provided critical feedback and helped shape the research, analysis, and manuscript.

## Conflicts of interest

The authors declare no conflict of interest.

## Supplementary Material

RA-014-D3RA07495A-s001

RA-014-D3RA07495A-s002
