# Supplementary material for: Synthesis and biological evaluation of novel 2-morpholino-4-anilinoquinoline derivatives as antitumor agents against HepG2 cell line
Source: RSC Adv. 2024 Jan 19;14(5):3304–13. doi: 10.1039/d3ra07495a (PMC10798140; doi:10.1039/d3ra07495a)

Electronic Supplementary Material (ESI) for RSC Advances.
This journal is © The Royal Society of Chemistry 2024

Synthesis and biological evaluation of novel 2-morpholino-4-anilinoquinoline derivatives as antitumor agents against HepG2 cell line

Ahmed Al-Sheikh[1], Malak A Jaber[1], Hana'a Khalaf[2], Nour AlKhawaja[3], Duaa Abuarqoub [4,5,*]

1 Department of Pharmaceutical Medicinal Chemistry and Pharmacognosy, Faculty of Pharmacy and Medical Sciences, University of Petra, Amman 11196, Jordan.

2 Department of Clinical Nutrition and Diets, Faculty of Pharmacy and Medical Sciences, University of Petra, Amman 11196, Jordan.

3 Pharmaceutical Studies Center, Faculty of Pharmacy and Medical Sciences, University of Petra, Amman 11196, Jordan.

4 Department of Pharmacology and Biomedical Sciences, Faculty of Pharmacy and Medical Sciences, University of Petra, Amman 11196, Jordan

5 Cell Therapy Center, University of Jordan, Amman 11942, Jordan

S1: Flow cytometry dot plots, analysis of apoptosis/necrosis cell death mechanism of 2-morpholino-4-anilinoquinoline derivatives on A) HepG2 and B) Fibroblast cells (NIH3T3) after treatment with Annexin V/PI dye. Cells of the chosen type were grown at a density of 2×10^5 cells/well for 72 hrs before being treated with one of the 2-morpholino-4-anilinoquinoline derivatives at two doses (IC_{50} and 20 µM). Control wells are untreated wells. The treated cells were then extracted and digested with Trypsin EDTA before being rinsed with phosphate-buffered saline and centrifuged at 300 x g for 5 min. The cell pellets were then stained with Annexin V/Propidium iodide stain as directed by the manufacturer (Invitrogen, Waltham, MA, USA). FACS DIVA 8 software and the FACSCanto™ II system (BD Biosciences, Franklin Lakes, NJ, USA) were used to analyze the samples immediately. Below is a comparison of a representative sample to an untreated control.

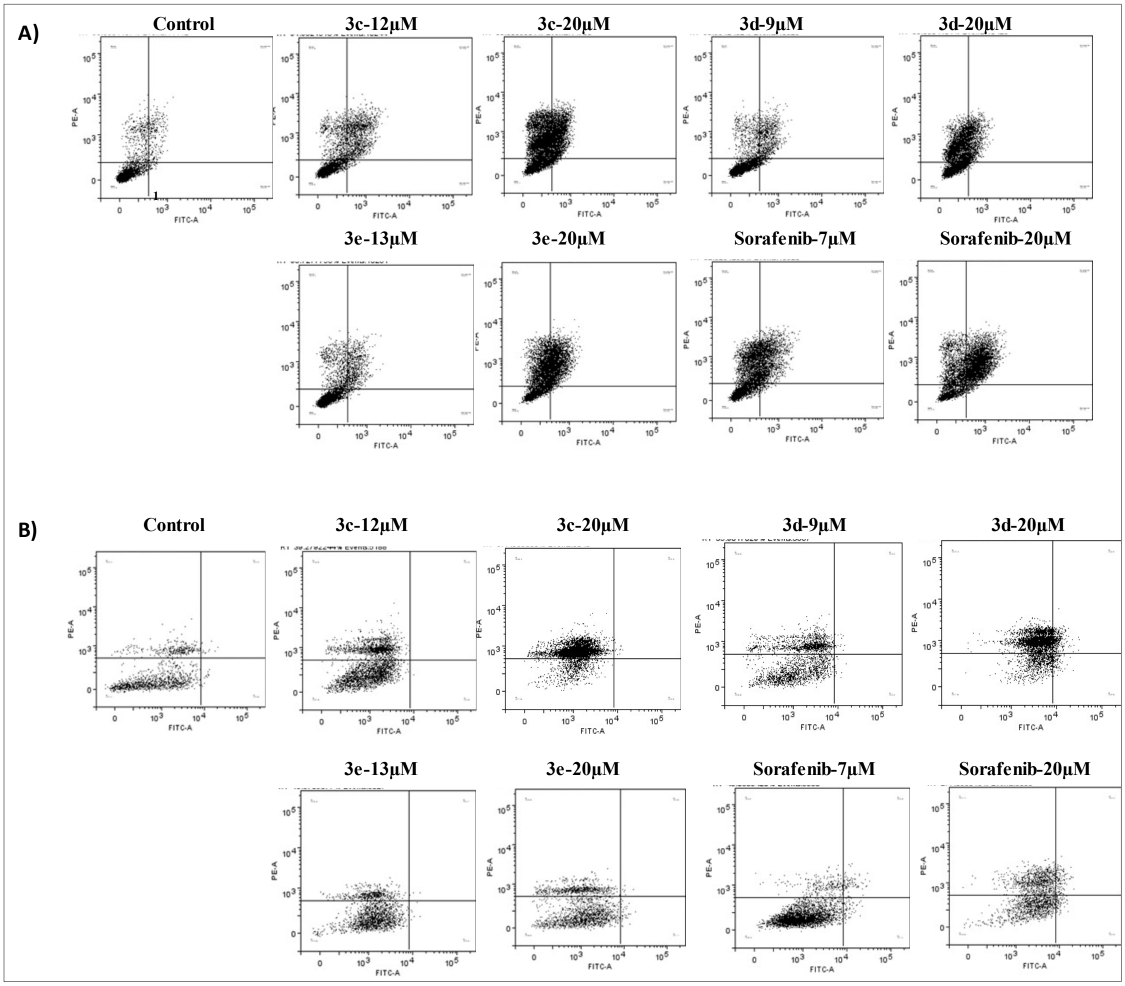

S2: Flow cytometry histograms comparing cell distribution within the cell cycle between control samples and treated cells. A uniparametric distribution of propidium iodide (PI)-stained cells previously treated with 2-morpholino-4-anilinoquinoline derivatives at two concentrations (IC_{50} and 20 µM) for 72 hrs prior to collection analyzed by Flow cytometry. Samples were examined by flow cytometry using the FACSCanto™ II system while data acquisition and analysis were performed by BD FACSDiva™ v8 and Flowlogic™ 7.3 using the cell cycle analysis module.

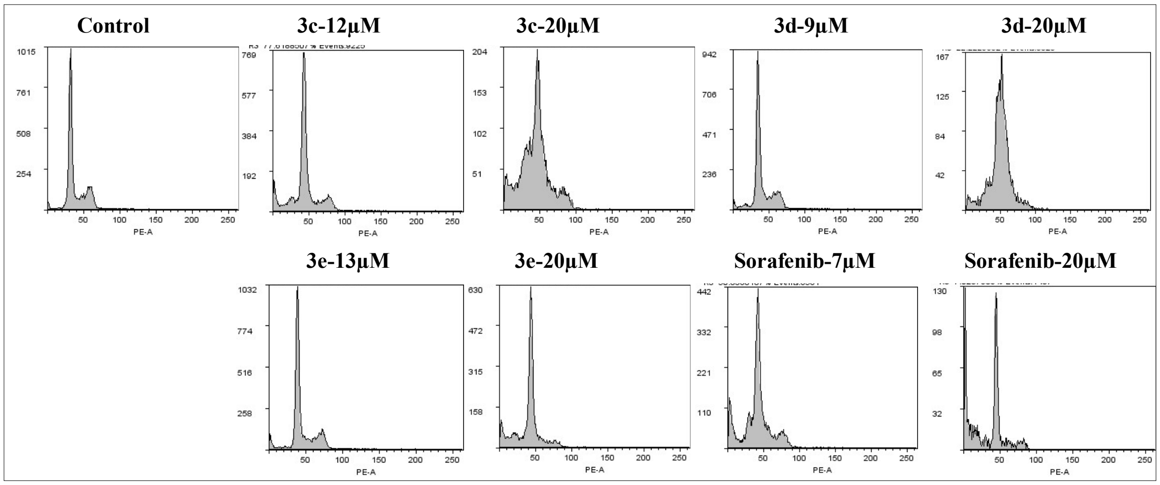

Supplement: RA-014-D3RA07495A-s001 [file RA-014-D3RA07495A-s001.pdf]
