# Supplementary material for: Synthesis and biological evaluation of novel 2-morpholino-4-anilinoquinoline derivatives as antitumor agents against HepG2 cell line
Source: RSC Adv. 2024 Jan 19;14(5):3304–13. doi: 10.1039/d3ra07495a (PMC10798140; doi:10.1039/d3ra07495a)

Electronic Supplementary Material (ESI) for RSC Advances.
This journal is © The Royal Society of Chemistry 2024

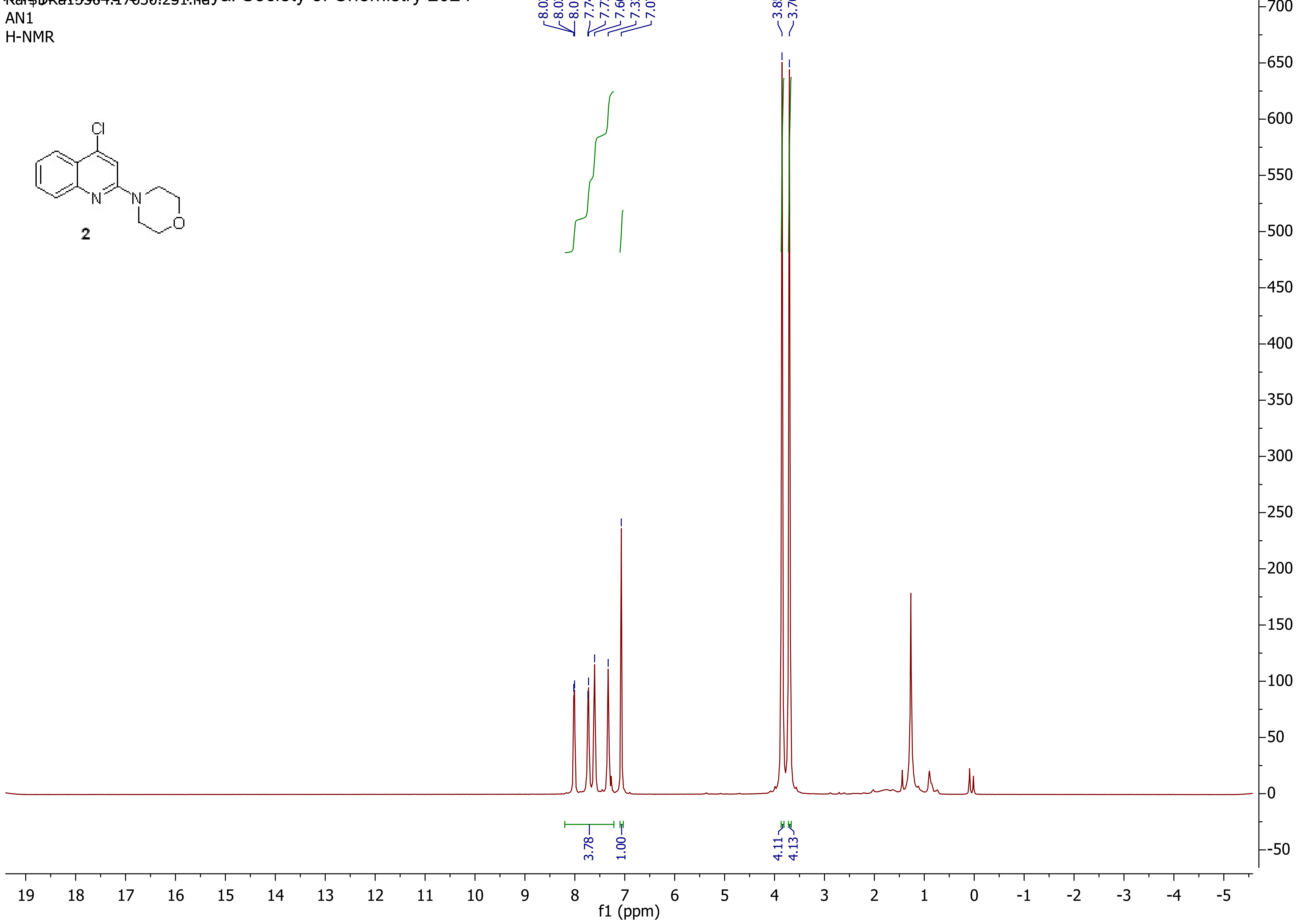

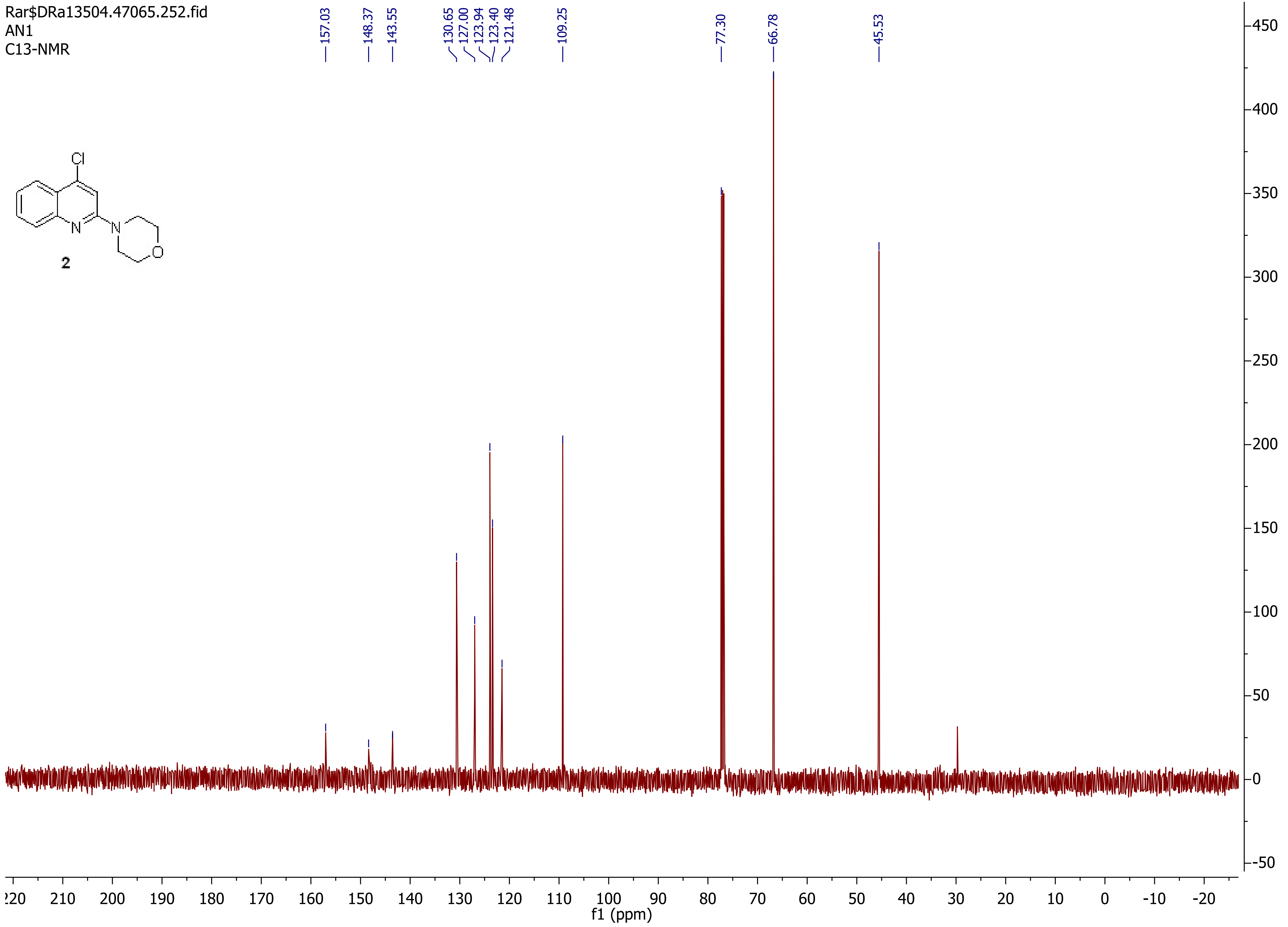
Rar$DRa13504.47065.252.fid
AN1
C13-NMR
157.03
148.37
143.55
130.65
127.00
123.94
123.40
121.48
109.25
77.30
66.78
45.53
Cl
N
N
O
2
f1 (ppm)

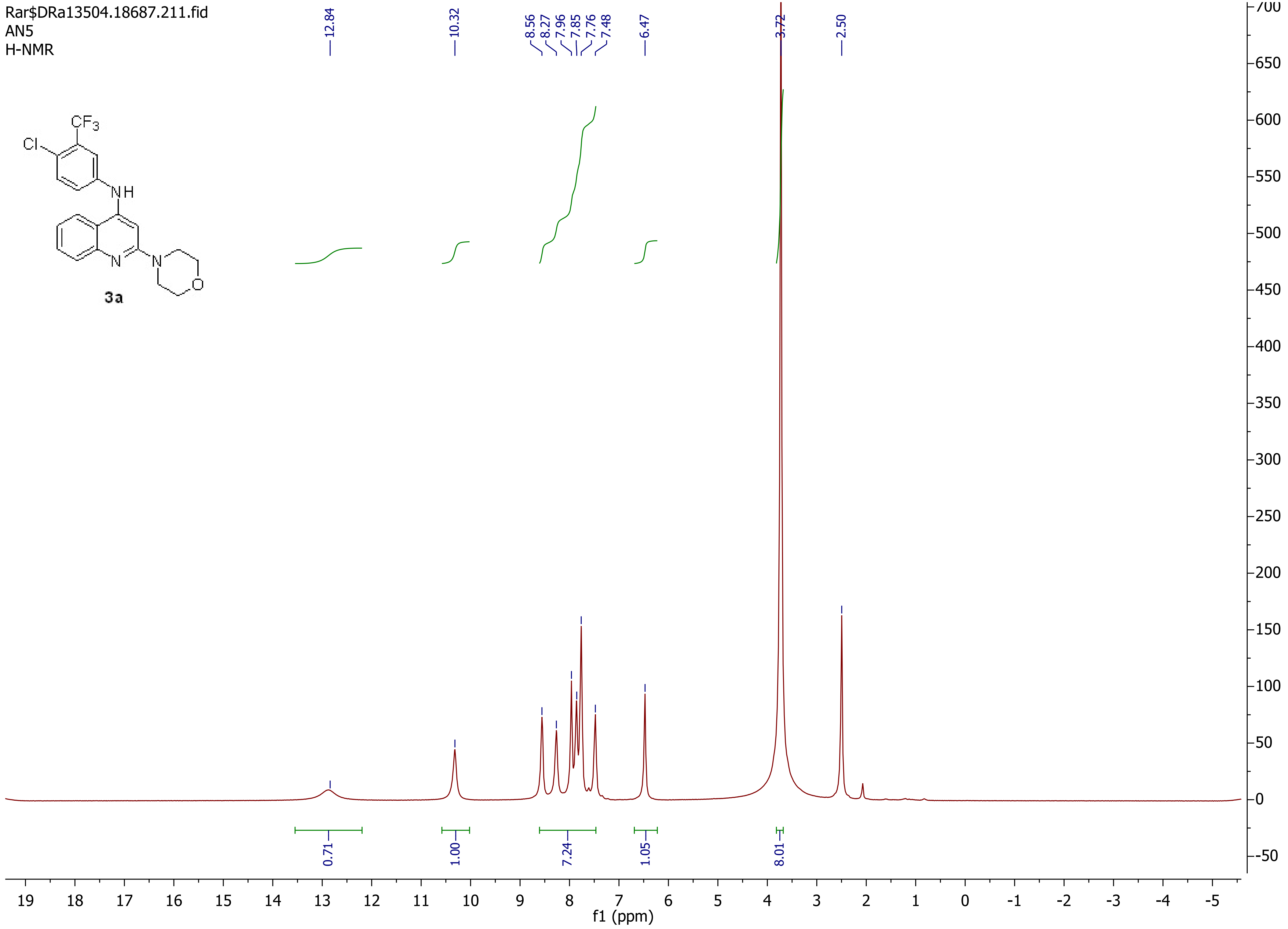
Rar$DRa13504.18687.211.fid
AN5
H-NMR
CF3
Cl
NH
N
N
O
3a
12.84
10.32
8.56
8.27
7.96
7.85
7.76
7.48
6.47
3.72
2.50
0.71
1.00
7.24
1.05
8.01
f1 (ppm)

Rar$DRa13504.45839.212.fid
AN5
C13-NMR

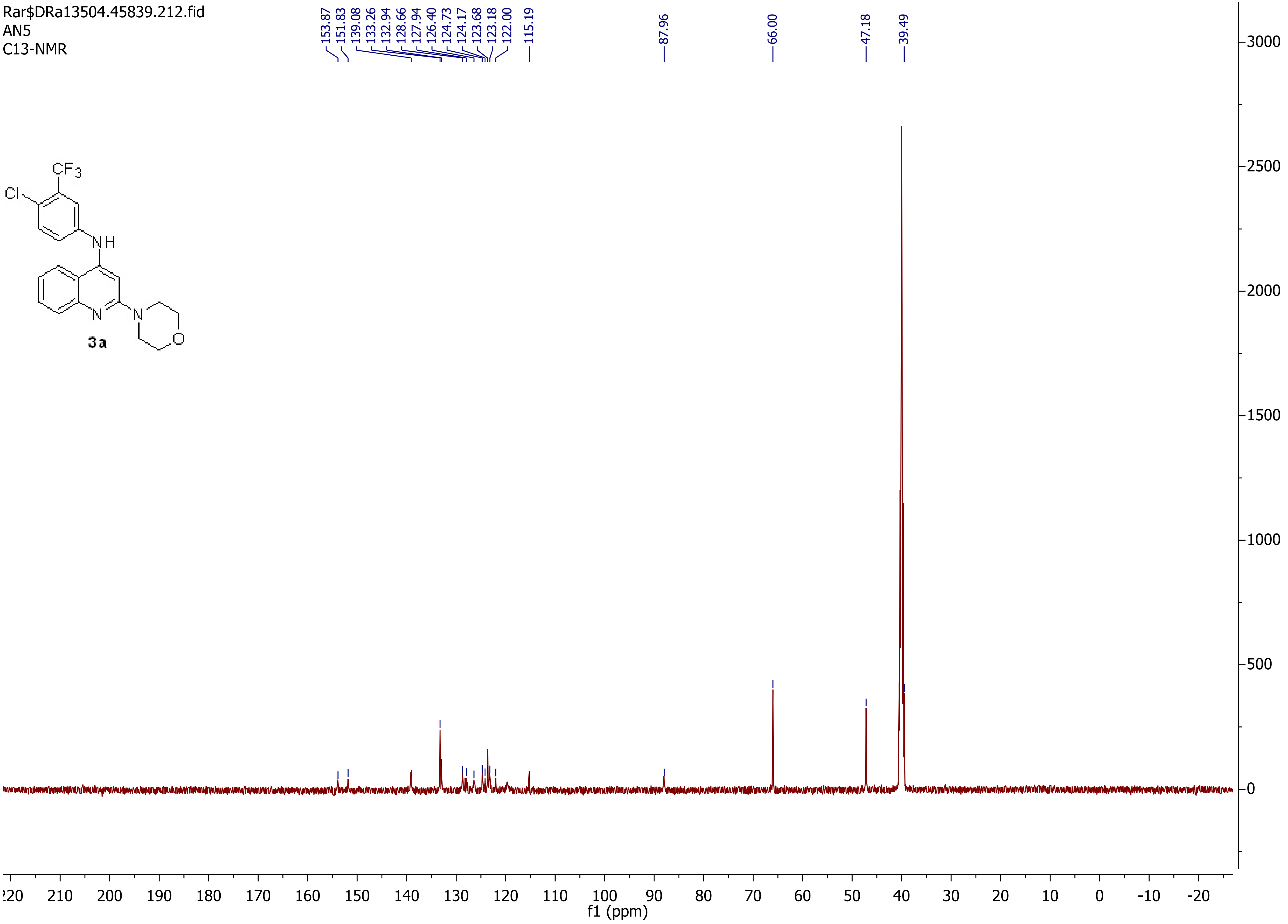

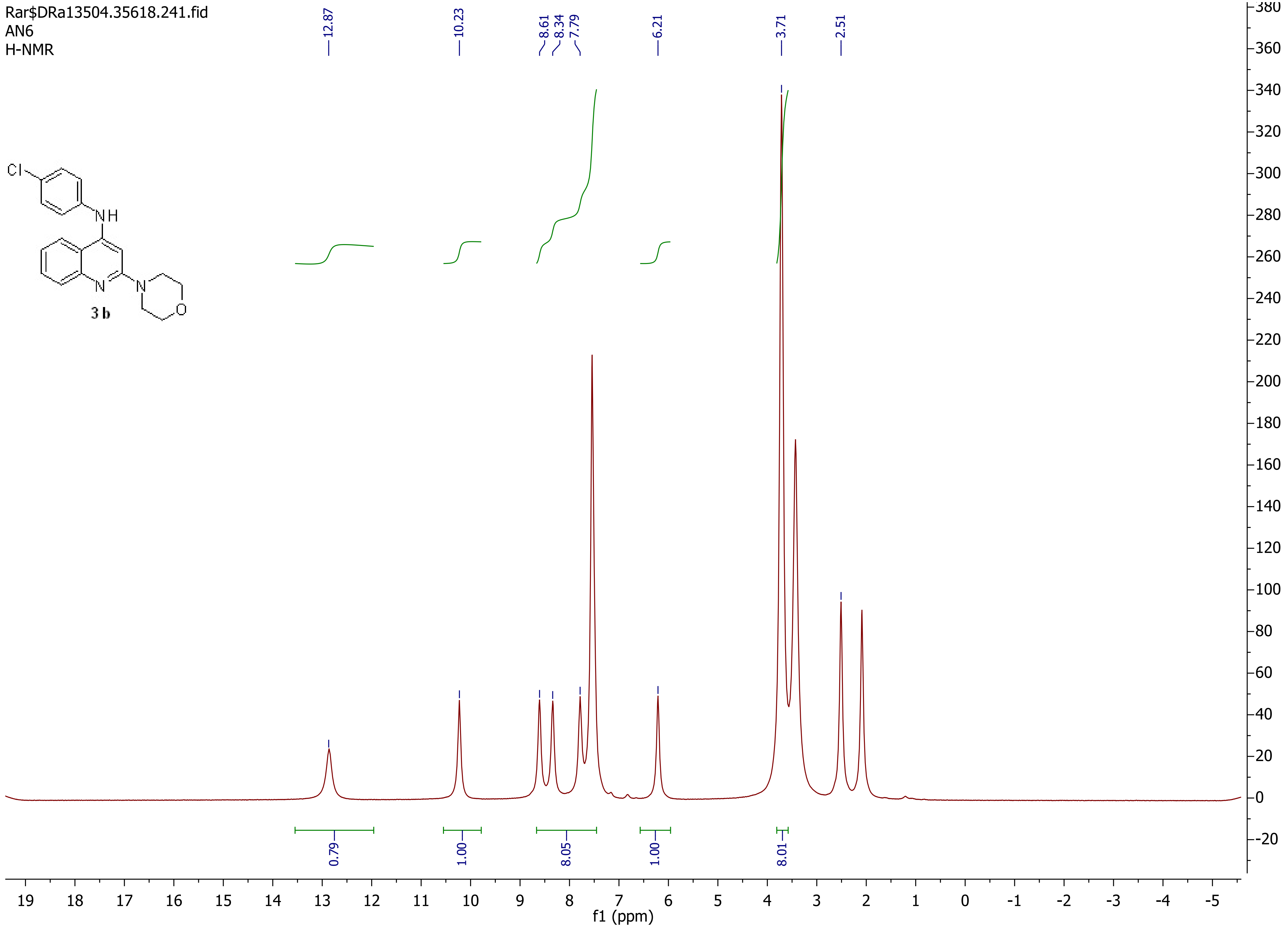

Rar$DRa13504.35618.241.fid
AN6
H-NMR
12.87
10.23
8.61
8.34
7.79
6.21
3.71
2.51
Cl
NH
N
N
O
3 b
0.79
1.00
8.05
1.00
8.01
f1 (ppm)

Rar$DRa13504.9844.242.fid
AN6
C13-NMR

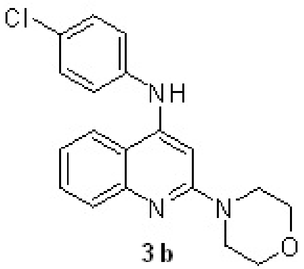

3 b

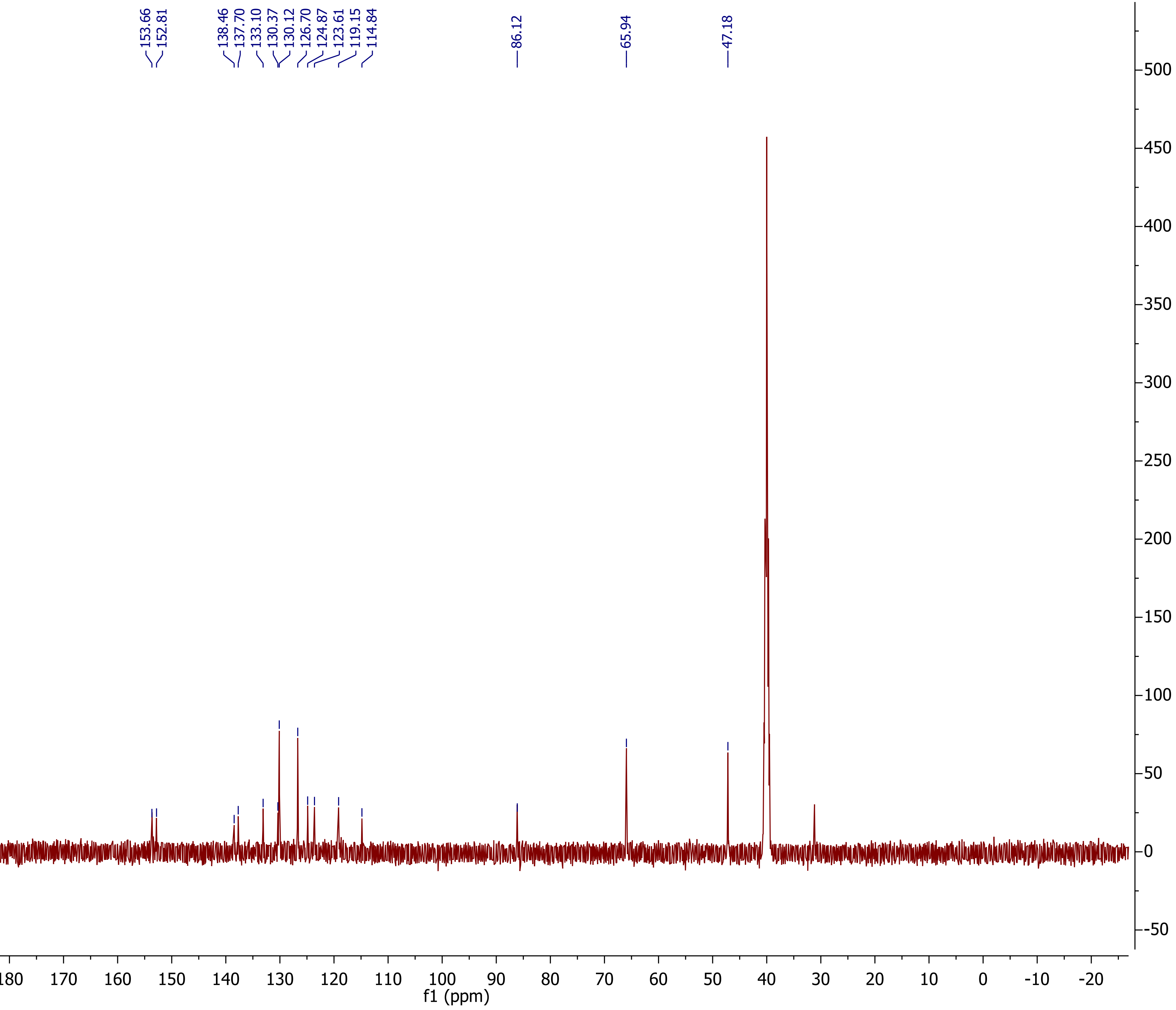

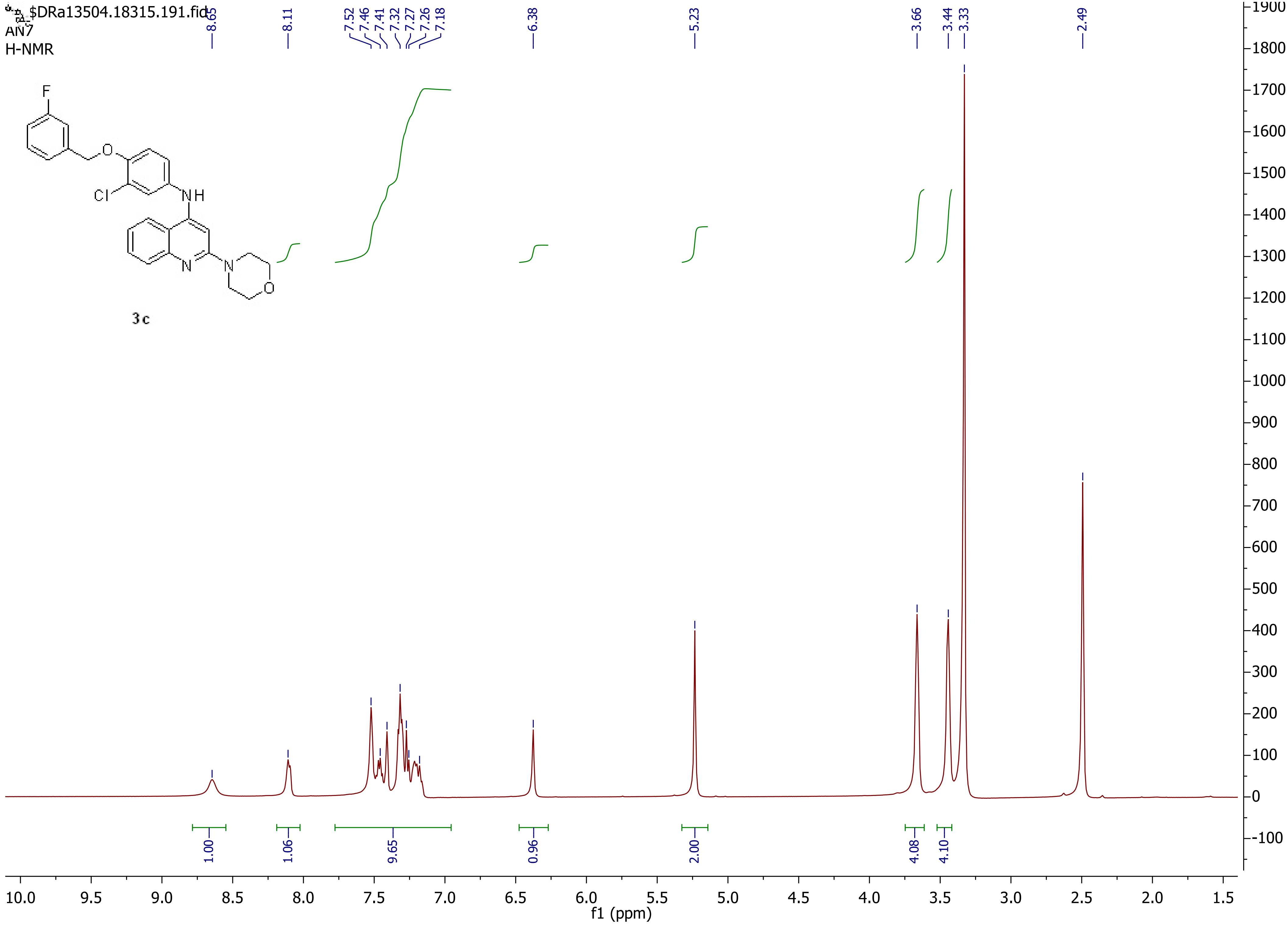
$DRa13504.18315.191.fid
AN7
H-NMR
8.65
8.11
7.52
7.46
7.41
7.32
7.27
7.26
7.18
6.38
5.23
3.66
3.44
3.33
2.49
F
O
Cl
NH
N
N
O
3c
1.00
1.06
9.65
0.96
2.00
4.08
4.10
f1 (ppm)

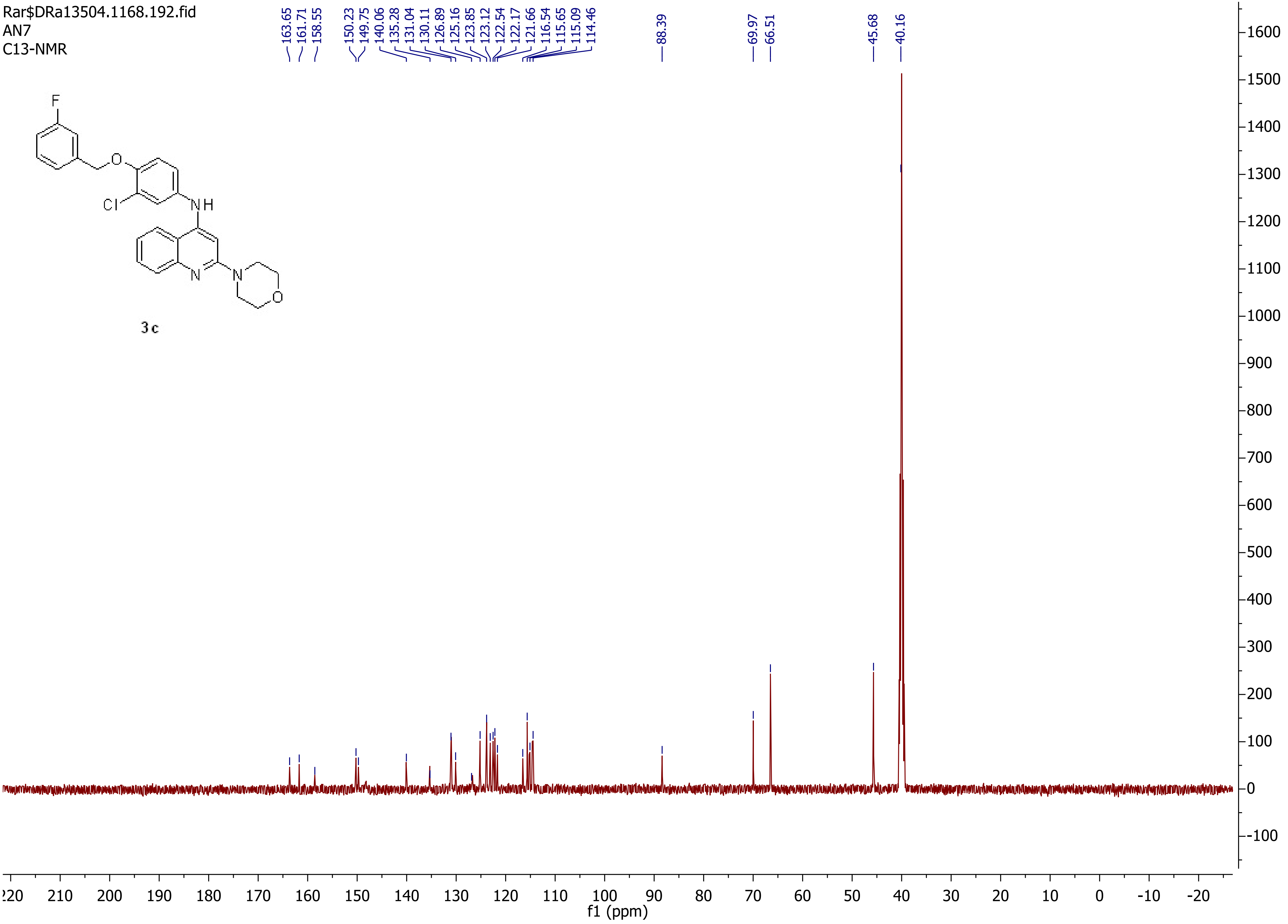

Rar$DRa13504.1168.192.fid
AN7
C13-NMR
163.65
161.71
158.55
150.23
149.75
140.06
135.28
131.04
130.11
126.89
125.16
123.85
123.12
122.54
122.17
121.66
116.54
115.65
115.09
114.46
88.39
69.97
66.51
45.68
40.16
F
O
Cl
NH
N
N
O
3c
f1 (ppm)

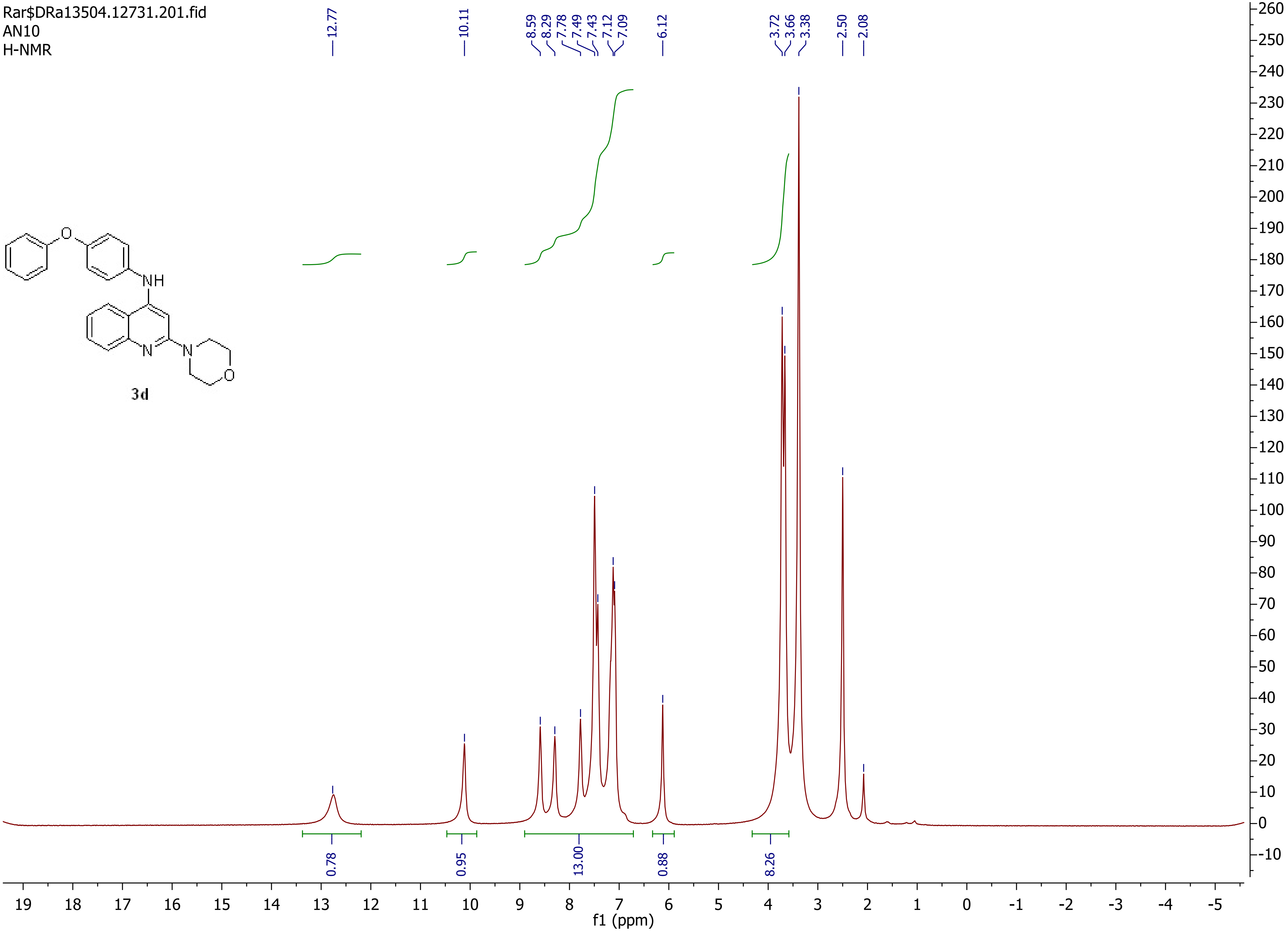

Rar$DRa13504.12731.201.fid
AN10
H-NMR
12.77
10.11
8.59
8.29
7.78
7.49
7.43
7.12
7.09
6.12
3.72
3.66
3.38
2.50
2.08
0.78
0.95
13.00
0.88
8.26
O
NH
N
N
O
3d
f1 (ppm)

Rar$DRa13504.11498.202.fid
AN10
C13-NMR

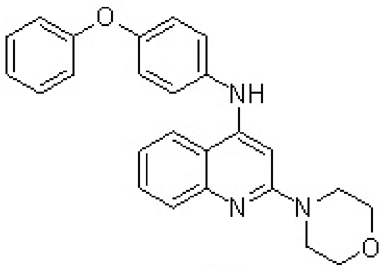

3d

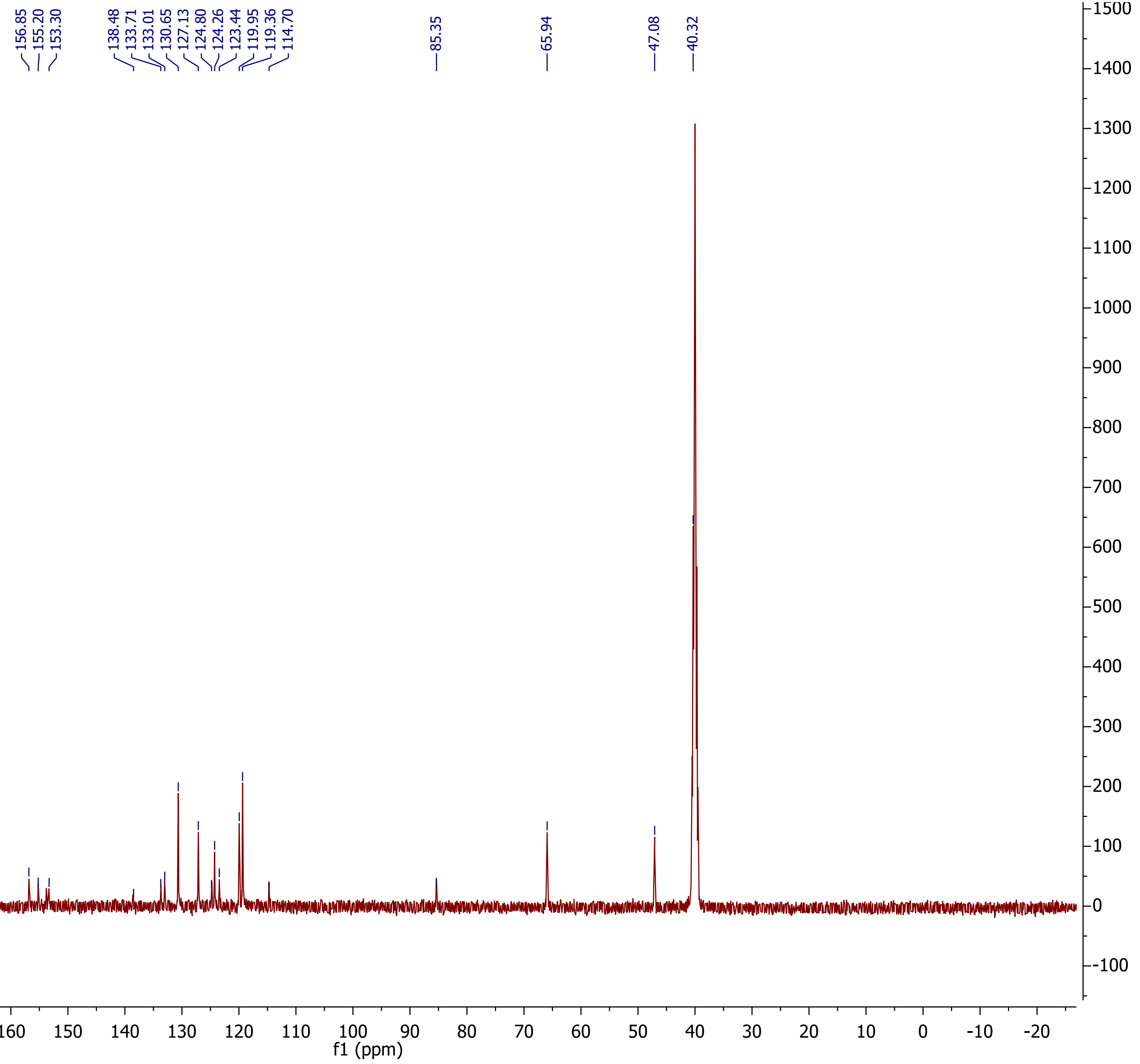

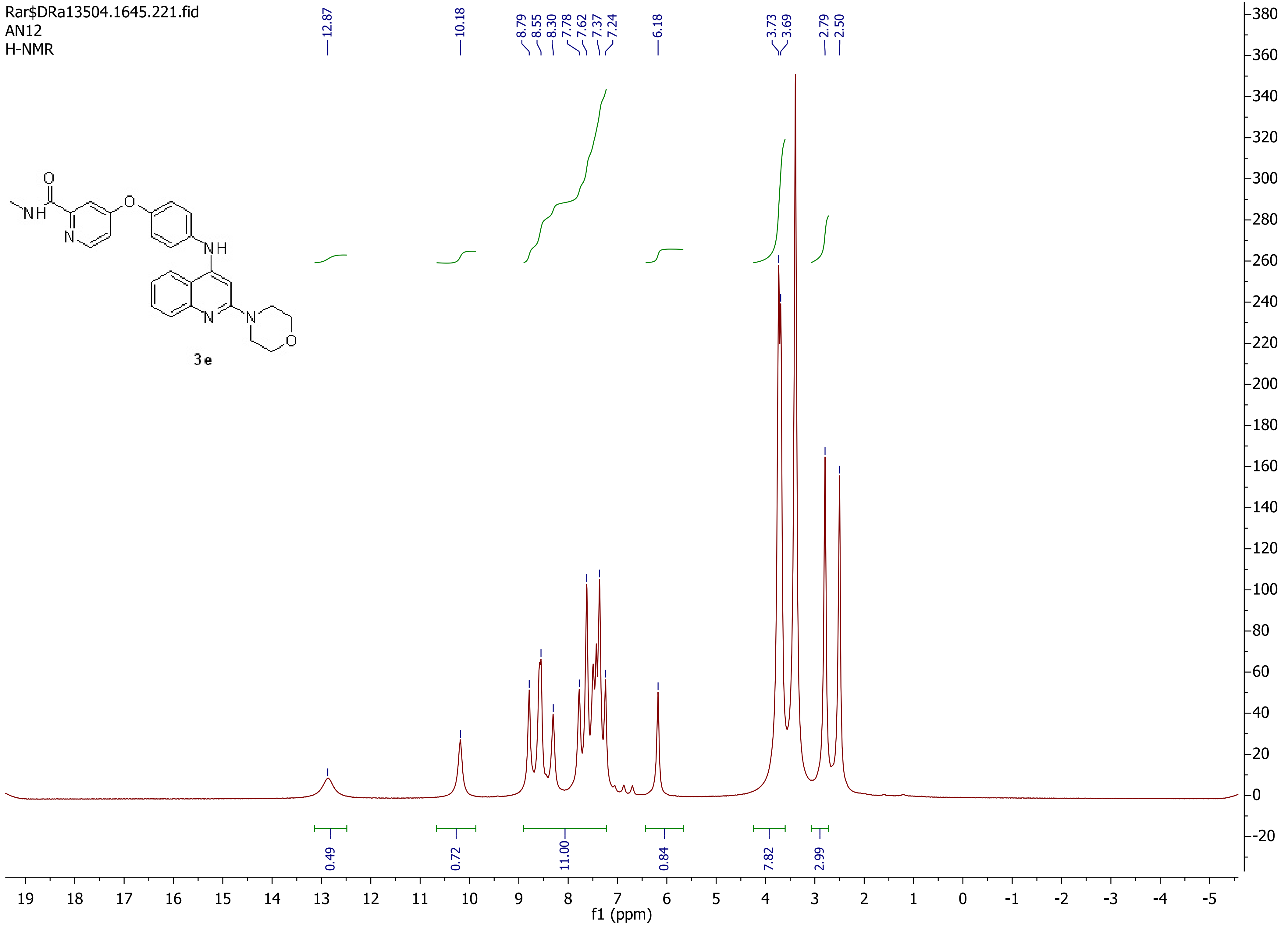

Rar$DRa13504.1645.221.fid
AN12
H-NMR
12.87
10.18
8.79
8.55
8.30
7.78
7.62
7.37
7.24
6.18
3.73
3.69
2.79
2.50
3e
0.49
0.72
11.00
0.84
7.82
2.99
f1 (ppm)

Rar$DRa13504.36190.222.fid
AN12
C13-NMR

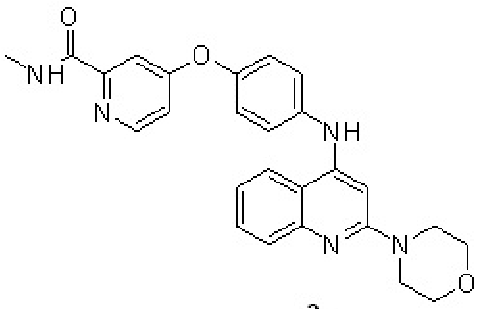

3e

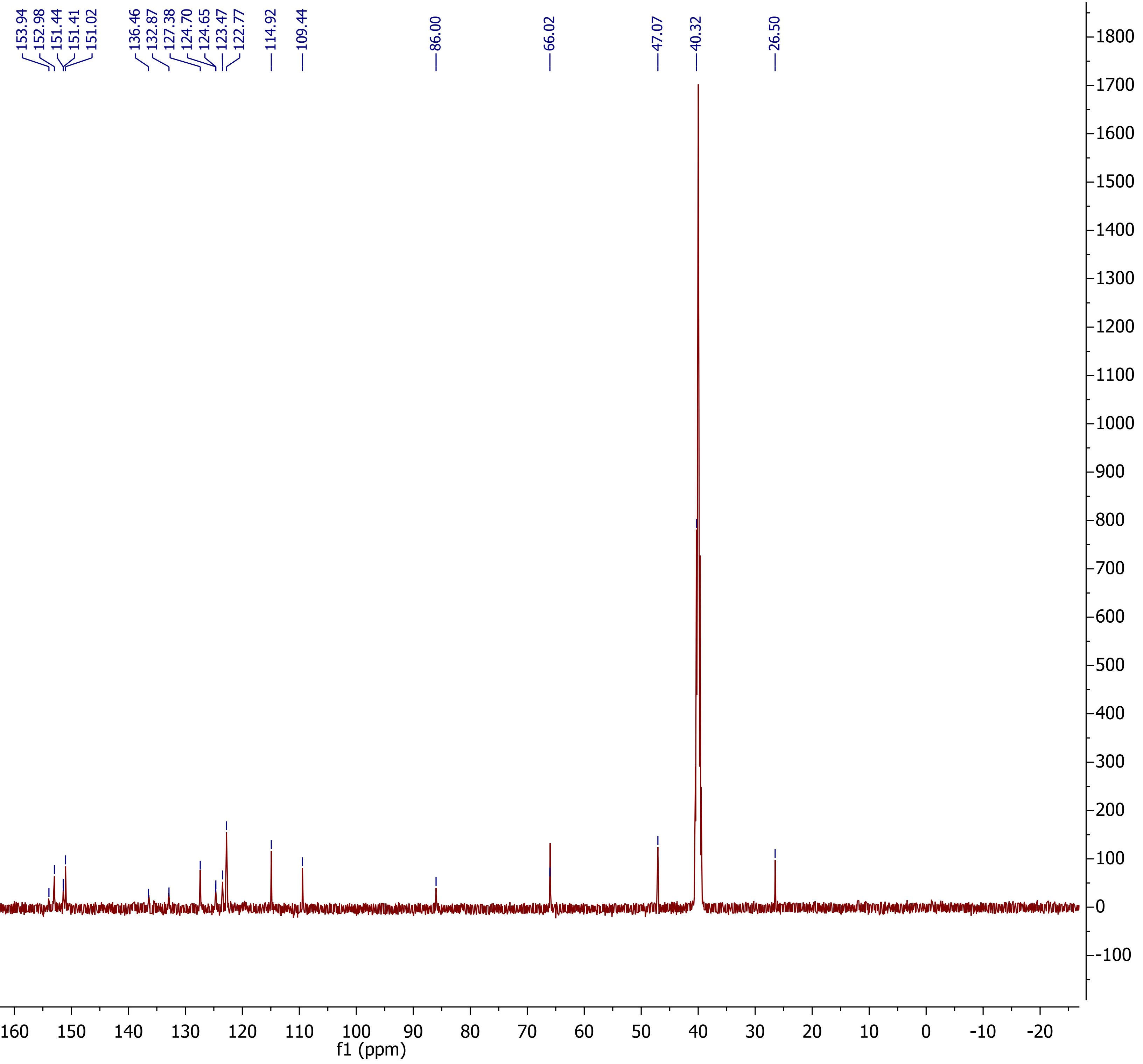

Supplement: RA-014-D3RA07495A-s002 [file RA-014-D3RA07495A-s002.pdf]
